# Brain Vital Signs: Expanding From the Auditory to Visual Modality

**DOI:** 10.3389/fnins.2018.00968

**Published:** 2019-01-18

**Authors:** Gabriela M. Pawlowski, Sujoy Ghosh-Hajra, Shaun D. Fickling, Careesa C. Liu, Xiaowei Song, Stephen Robinovitch, Sam M. Doesburg, Ryan C. N. D'Arcy

**Affiliations:** ^1^NeuroTech Laboratory, Faculty of Applied Sciences, Simon Fraser University, Burnaby, BC, Canada; ^2^Biomedical Physiology and Kinesiology, Faculty of Science, Simon Fraser University, Burnaby, BC, Canada; ^3^Health Sciences and Innovation, Surrey Memorial Hospital, Fraser Health, Surrey, BC, Canada

**Keywords:** electroencephalogram (EEG), event-related potentials (ERPs), clinical assessment, neurology, point-of-care, vital signs

## Abstract

The critical need for rapid objective, physiological evaluation of brain function at point-of-care has led to the emergence of brain vital signs—a framework encompassing a portable electroencephalography (EEG) and an automated, quick test protocol. This framework enables access to well-established event-related potential (ERP) markers, which are specific to sensory, attention, and cognitive functions in both healthy and patient populations. However, all our applications to-date have used auditory stimulation, which have highlighted application challenges in persons with hearing impairments (e.g., aging, seniors, dementia). Consequently, it has become important to translate brain vital signs into a visual sensory modality. Therefore, the objectives of this study were to: 1) demonstrate the feasibility of visual brain vital signs; and 2) compare and normalize results from visual and auditory brain vital signs. Data were collected from 34 healthy adults (33 ± 13 years) using a 64-channel EEG system. Visual and auditory sequences were kept as comparable as possible to elicit the N100, P300, and N400 responses. Visual brain vital signs were elicited successfully for all three responses across the group (N100: *F* = 29.8380, *p* < 0.001; P300: *F* = 138.8442, *p* < 0.0001; N400: *F* = 6.8476, *p* = 0.01). Initial auditory-visual comparisons across the three components showed attention processing (P300) was found to be the most transferrable across modalities, with no group-level differences and correlated peak amplitudes (rho = 0.7, *p* = 0.0001) across individuals. Auditory P300 latencies were shorter than visual (*p* < 0.0001) but normalization and correlation (*r* = 0.5, *p* = 0.0033) implied a potential systematic difference across modalities. Reduced auditory N400 amplitudes compared to visual (*p* = 0.0061) paired with normalization and correlation across individuals (*r* = 0.6, *p* = 0.0012), also revealed potential systematic modality differences between reading and listening language comprehension. This study provides an initial understanding of the relationship between the visual and auditory sequences, while importantly establishing a visual sequence within the brain vital signs framework. With both auditory and visual stimulation capabilities available, it is possible to broaden applications across the lifespan.

## Introduction

There is an increasing need for objective, neurophysiological measures, such as EEG, to provide unbiased measures of brain function across a range of different points-of-care. In terms of deployable technologies, EEG benefits from being low-cost, non-invasive, and is particularly well-suited for clinical applications (Connolly et al., 1995; D'Arcy et al., 2003; Gawryluk et al., 2010; Giacino et al., 2014; Sculthorpe-Petley et al., 2015; Ghosh-Hajra et al., 2016a; Fickling et al., 2018). From EEG, a range of markers indexing information processing from low-level sensory to higher-level cognitive processing can be extracted as event-related potentials (ERPs) reflecting underlying sensory, attentional, cognitive processing (D'Arcy et al., 2000; Gawryluk et al., 2010). The translation of EEG/ERP research into neurophysiological assessment applications compatible with the clinical environment has been demonstrated with rapid non-invasive implementations, such as the Halifax Consciousness Scanner (HCS; D'Arcy et al., 2011) and more recently in the brain vital signs framework (Ghosh-Hajra et al., 2016a). Typically ERPs are studied individually using lengthy testing times. However, the brain vital signs framework combines well-established methods utilizing a rapid, integrated, and fully automated ERP stimulation sequence to elicit three targeted ERP responses. A results report is generated based on normalized ERP characteristics. This has been validated in large samples of healthy individuals by reliably eliciting the targeted ERPs across individuals (Ghosh-Hajra et al., 2016a). Changes in these targeted ERPs have been observed in patients with acquired brain injuries (Fleck-Prediger et al., 2014) and athletes with concussions (Fickling et al., 2018).

The brain vital signs framework focuses on three well-established ERPs: (1) the N100 reflecting sensory processing (Davis, 1939); (2) the P300 reflecting attention processing (Sutton et al., 1967); and N400 reflecting semantic/language processing (Kutas and Hillyard, 1980). Individual-level results evaluate response amplitudes and latencies compared to a normative dataset, to form Elemental Brain Scores (EBS) (Ghosh-Hajra et al., 2016a). EBS comparisons are a linear transformation into standardized and normalized scores ranging from 0 to 1, ranked based on the range in the normative group (Ghosh-Hajra et al., 2016a). Therefore larger response amplitudes and shorter response latencies result in higher scores for each of the three ERP responses (3 responses ^*^ 2 metrics = 6 EBS). Importantly, EBS results enable standardization across different modalities and acquisition systems. EBS results can then be presented graphically on a radar plot to provide a simple output with a typically normative hexagonal shape (**Figure 4**).

The auditory brain vital signs stimulus sequence utilizes an interlaced design to elicit the three ERPs in parallel and optimize the number of trials per unit time, therefore avoiding the traditionally lengthy serial testing procedures (see Ghosh-Hajra et al., 2016a). The auditory stimulus sequence consists of a passive auditory oddball paradigm and spoken word pairs. The oddball paradigm includes tones divided into standard and deviant conditions, where the N100 and P300 components are derived from the deviant condition. Prime-target word pairs are divided into congruent (e.g., bread-butter), and incongruent (e.g., bread-window) pairs. The N400 is derived from the incongruent word pairs and shows comparable features to the conventional semantic N400 (Ghosh-Hajra et al., 2016a, 2018).

To date, brain vital sign applications have been developed using the auditory sensory modality (Ghosh-Hajra et al., 2016a; Fickling et al., 2018). However, as the aging population grows (Grenier, 2017) there will be an increasing demand for accessibility to objective testing of cognitive function, such as with brain vital signs. The adaptation to a visual modality will address critical limitations around hearing loss and impairments in aging populations and enable wider application across the lifespan. Accordingly, the aim of this study was to expand the brain vital signs application by translating the established brain vital signs auditory test into a visual test to elicit similar targeted ERP responses.

### Translation From the Auditory to Visual Modality

The established auditory brain vital signs sequence structure can easily be adapted into the visual modality by utilizing previous research on the well-established visual ERPs: N100, P300, and N400. Previous studies have successfully utilized a simple visual oddball paradigm using brightness of stimuli to elicit the visual N100 (Johannes et al., 1995; Polich et al., 1996; Carrillo-de-la-Peña et al., 1999). A more recent comparison study used changing black and white full-view flashes in both an active (counting) and passive (no counting) task to evoke and record a frontal-central N100 (Huang et al., 2011). The anterior N100 subcomponent typically occurs around 80-150ms and is best recorded at frontal and central electrode sites (Fz and Cz), similar to the auditory N100 (Vogel and Luck, 2000; Knott et al., 2003; Huang et al., 2011).

Similarly, the P300 response has typically been elicited within the visual modality by randomly changing physical visual characteristics, such as colors, shapes, letters, words, or pictures (Comerchero and Polich, 1998; Bennington and Polich, 1999; Bernat et al., 2001; Bledowski, 2004; Cano et al., 2009; Duncan et al., 2009; Kappenman and Luck, 2012, pp.159-180; Mertens and Polich, 1997; Stevens et al., 2000; Knott et al., 2003). A robust P300 response has also been observed to a particularly relevant and salient stimulus, such as a subject's own name (SON) when presented with low probability (see review of SON paradigms: Berlad and Pratt, 1995; Perrin et al., 1999, 2006). When presented visually, the SON response has shown an enhanced P300 response at central electrodes compared to other similar or differing stimuli within a 350–850 ms interval (Zhao et al., 2009, 2011; Cygan et al., 2014; Tacikowski and Nowicka, 2010). Besides being particularly salient, SON paradigms also have benefits for a rapid, visual sequence, because it has been found to be particularly resistant to repetition blindness during rapid serial visual presentations (Arnell, 2006; Tacikowski and Nowicka, 2010).

Lastly, the N400 can be readily elicited by visual word pair paradigms involving violations of semantic expectancies (Kutas and Hillyard, 1982; Bentin et al., 1985; Rugg, 1985; Brown and Hagoort, 1993; Kutas and Van Petten, 1994; Chwilla et al., 1998; D'Arcy and Connolly, 1999; Brown et al., 2000; D'Arcy et al., 2005; Lau et al., 2008). The N400 is typically found between 200 and 600 ms post-stimulus (Kutas and Federmeier, 2011; Ghosh-Hajra et al., 2018), irrespective of the modality, with maximal amplitudes at midline central or parietal sites and noticeably smaller amplitudes at prefrontal and lateral frontal sites (Duncan et al., 2009). We recently reported a functional neuroimaging study using magnetoencephalography (MEG) that confirmed similar neuroanatomical correlates for the N400, which is the latest and highest-level ERP component within the brain vital signs framework (Ghosh-Hajra et al., 2018).

### Objectives

This study aimed to develop and validate a visual brain vital signs sequence on healthy adults to increase accessibility for individuals with hearing impairments. This challenge has been identified frequently as a central issue for developing brain vital sign monitoring in age-related cognitive impairment and dementia, in which hearing loss can be a major barrier. There were two main objectives:

1. Translate the brain vital signs framework into a visual version and validate the new sequence by assessing if the targeted ERPs (N100, P300, and N400) were evoked successfully; and
2. Compare the ERP responses (amplitudes and latencies) between visual and auditory modalities, and evaluate the relationship between modalities within individuals.

## Methods

### Participants

Thirty-four (34) healthy, adult participants were enrolled in the study (mean age: 33 ± 13 years, 16 females). Informed consent was given from each participant. Participants had no history of neurological problems or psychoactive medications. All individuals were fluent in English and had normal or corrected-to-normal vision and hearing. The Research Ethics Boards at Simon Fraser University and Fraser Health Authority approved the study.

### Stimulus Sequence

The stimulus sequence was adapted from previous brain vital signs studies which utilizes an interlaced structure with an oddball paradigm and word pair paradigm (Ghosh-Hajra et al., 2016a). An oddball paradigm consists of frequent, standard stimuli and deviant, rare stimuli conditions. The oddball paradigm was split into 67% standard and 33% deviant, with the N100 and the P300 derived from the deviant condition. The 72-paired words were divided into congruent prime pairs (e.g., romeo-juliet, 50%) and incongruent prime pairs (romeo-coffee, 50%). The N400 was derived from the incongruent words condition. Both sequences were passive tasks (no response required). The auditory sequence consisted of tones (250 ms duration, standard 75 dB tones, deviant 100 db tones), and spoken word pairs (~1000 ms duration).

**Figure 1:**
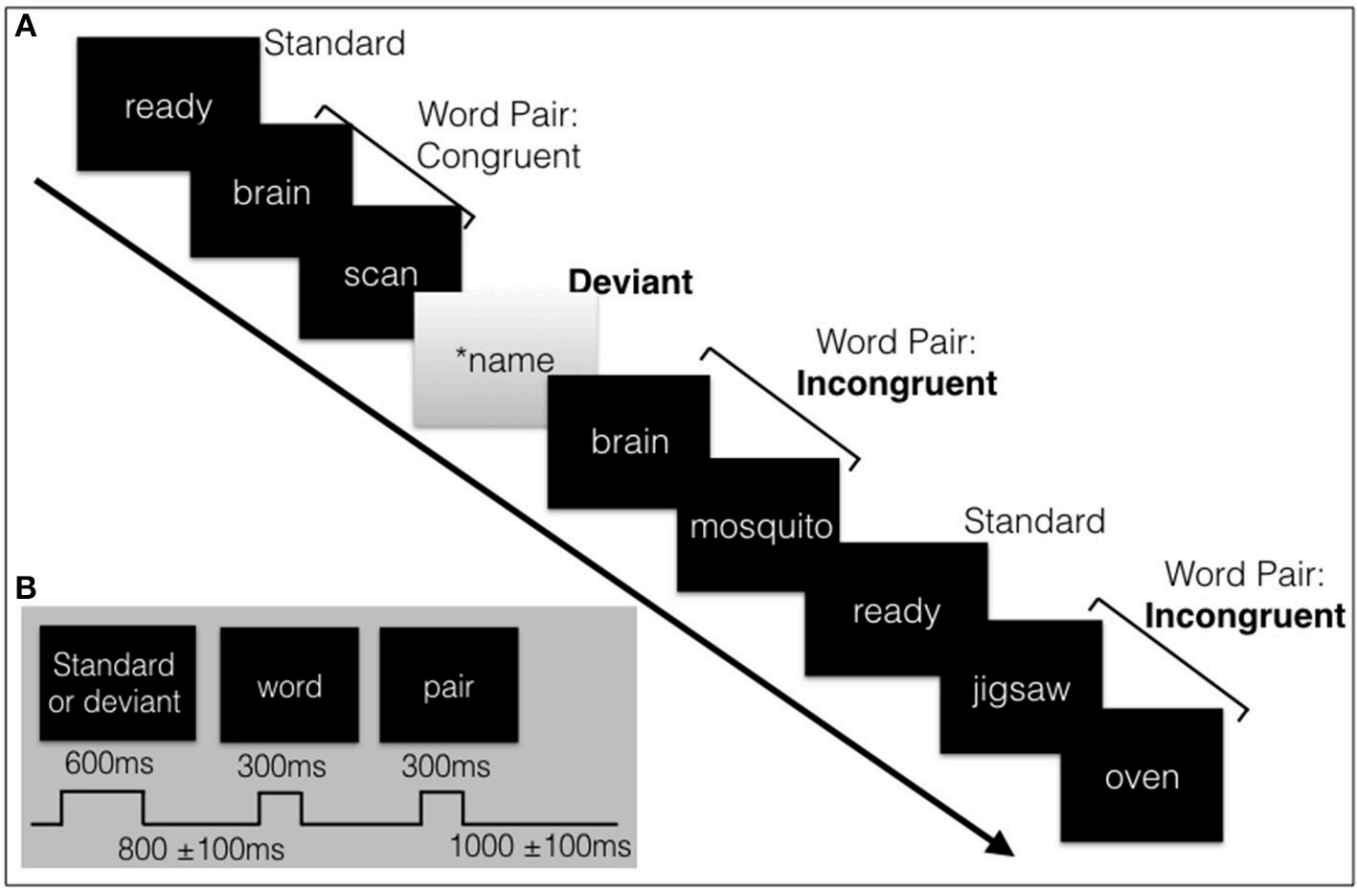
**(A)** Schematic illustration of a sample of the visual stimulus sequence, containing the subjects' name, and word pairs. **(B)** The length of the stimuli and inter-stimulus intervals with jitter. Total sequence is around 4.6 minutes in length.

The interlaced structure of the visual stimulus was designed to be similar to that of the auditory sequence; a 4.6 min interlaced oddball and word pair sequence (see Figure 1). The level of intensity and difficulty of the auditory and visual needed to be matched because such factors can affect the amplitude and latency of components, particularly the P300 in a passive task. A response does not add much value for the N100 (sensory processing) and N400 (sematic processing) (Kappenman and Luck, 2012, pp. 397–440) but does affect the P300 (attention processing). When compared to active tasks, the passive oddball paradigm in both modalities has shown reduced amplitudes (Bennington and Polich, 1999). Nonetheless, passive paradigms have still shown highly comparable and reliable P300 responses (Polich and McIsaac, 1994). A passive task is preferred for patient populations that may struggle with responses or demanding tasks, such as young children or dementia patients (Perrin et al., 1999; Marchand et al., 2002; Huang et al., 2011; Sculthorpe-Petley et al., 2015; Ghosh-Hajra et al., 2016b, 2018; Hajra et al., 2018). Based on past research, a salient passive visual task, a contrast flip and SON, was chosen to ensure a N100 response and a robust visual P300 response. Another advantage of a passive task is that it requires much less time than an active task which requires time for a response, and also greatly reduces the potential for unnecessary muscle movement artifact to the EEG data collection.

All visual stimuli were presented serially in the center of the screen. The words were presented in white font (Sans serif, size 56) on a black background. The standard (“ready”) or deviant (SON in inverse contrast) had a duration of 600 ms followed by the prime and target words pairs, duration of 300 ms each. A random jitter was incorporated into the inter-stimulus-interval (ISI) (800 ms ± 100 ms) and in the inter-block interval (IBI) (1000 ms ± 100 ms) to avoid repetition blindness, habituation, and potential entrainment of alpha rhythm with the stimulus timing which can affect the amplitude and/or latency of components and quality of the data (Luck, 2014, pp. 203–204; Ravden and Polich, 1998).

### EEG Data Acquisition

Each participant was assessed with both visual and auditory brain vital sign versions, using a counterbalanced order across participants. Data were collected in a dedicated EEG room with consistent conditions (i.e., brightness) across participants. Visual stimuli were presented on a computer monitor centered 75 cm in front of the participant. Acoustic stimuli were delivered binaurally through insert headphones, with participants maintaining visual fixation on a cross displayed in the center of the screen. Both the auditory and visual sequences were delivered using Presentation® software (Version 18.0, Neurobehavioral Systems, Inc., Berkeley, CA, www.neurobs.com). All EEG data were recorded using a 64-channel EEG system using active Ag/AgCl electrodes (BrainAmp 64-channel system actiCAP). Raw EEG data were recorded by BrainVision Recorder (Version 1.20.0801 Brain Products GmbH). The impedance for each electrode within the 64-channel cap was maintained below 20 kΩ; it was checked at the start of data collection and in the breaks between runs.

### EEG Pre-processing and ERP Analysis

EEG analysis was done using Brain Vision Analyzer® software, version 2.03 (Brain Products, Gilching, Germany). EEG data were down-sampled from 1000 to 500 Hz. All 64- channels were inspected for noise and re-referenced offline from the BrainVision Recorder's own initial reference channel, FCz, to the average of the two mastoids (electrodes TP9 and TP10), We chose this after careful consideration in literature and for compatibility with other bimodal comparison studies (Huang et al., 2011; Campanella et al., 2012; Dreo et al., 2017; Holcomb et al., 1992). A 0.1–50 Hz zero phase-shift, 4th order Butterworth bandpass filter and 60 Hz notch filter was applied to the data. EEG data were segmented into epochs from −100 to 900 ms time-locked to stimulus onset. Artifact rejection was done using gradients (maximal allowed voltage step: 10 uV/ms and maximal allowed difference of values in intervals: 100 uV), and visually reviewed for each subject. In line with prior work (Liu et al., 2017, 2018), independent component analysis (ICA) was performed for artifact correction (e.g., blinks, saccades, cardiac activity, muscle contractions, breathing) using the Infomax algorithm (Lee et al., 1999). Segments were baseline corrected (−100 to 0 ms), low-passed filtered at 20 Hz, and averaged based on experimental condition (Luck, 2014). Data from four participants were excluded due to EEG noise and task compliance issues.

#### Targeted ERP Responses: Mean Amplitude Analysis

Mean amplitude analysis was chosen to address Objective 1. Mean amplitude measures were used in order to avoid selection bias when first establishing the sequence (Objective 1) (Luck, 2014, pp. 285–290). This method is also advantageous because conditions with differing number of trials (i.e., standard and deviant) or noise levels (i.e., artifacts) do not affect the results, allowing for all trials to be kept, providing greater statistical power (reducing Type I error rate). Mean amplitude analysis was done using MATLAB (Mathworks, USA) and ERPLAB, an open-source Matlab package (Lopez-Calderon and Luck, 2014). Mean amplitudes were calculated for each stimulus type for each individual at 3 midline electrode sites (Fz, Cz, and Pz). Each latency window was guided by past literature recommendations and visual inspection of the grand average (GA) waveforms (Chronaki et al., 2012; Pfabigan et al., 2014). The N100 was indexed by differential activity within a 50 ms window, as recommended for early components (Vogel and Luck, 2000; Luck, 2014, pp. 286–287). The P300 was measured over a 200 ms window (Wood et al., 2006; Cano et al., 2009). The N400 was measured over a shorter latency for visual (400 ms) than auditory (500 ms), because the visual N400 is typically shorter in duration compared to the auditory N400 (Kutas and Van Petten, 1994; Kutas and Federmeier, 2011). Mean amplitudes were calculated over the following latency windows for the auditory data: 114–164 ms (N100), 250–450 ms (P300), and 200–700 ms (N400). The indexed windows chosen for measuring mean amplitudes in the visual data were: 87–137 ms (N100), 300–500 ms (P300), and 200–600 ms (N400).

Statistical analysis was performed using JMP (JMP®, Version 12.2.0 SAS Institute Inc., Cary, NC). Normality was assessed using the Shapiro-Wilk W test. To assess the difference between stimulus types, a repeated-measures ANOVA was used with the mean amplitude values for each component within each modality, with two factors: stimulus (standard vs. deviant or congruent vs. incongruent) and electrode site (Fz, Cz, and/or Pz). The number of levels for site was specific to each component based on previously reported maximal sites; frontal-central channels (Fz and Cz) were chosen for N100 (Vogel and Luck, 2000; Knott et al., 2003; Huang et al., 2011), central sites (Fz, Cz, and Pz) were chosen for P300 (Zhao et al., 2009, 2011; Tacikowski and Nowicka, 2010; Cygan et al., 2014) and central-parietal (Cz and Pz) for the N400 (Duncan et al., 2009). Greenhouse-Geisser adjusted values were used to correct for any violations of sphericity assumptions. Student *t*-tests with Tukey-Kramer correction for multiple comparisons were applied for all *post-hoc* comparisons to adjust alpha levels. For data that did not pass the Shapiro-Wilk W test of normality, the Wilcoxon signed-rank test was used.

#### Comparison and Normalization of Auditory and Visual Sequences: Adjusted Baseline Amplitude and Peak Latency Measures

Once the targeted components were confirmed using mean amplitude analysis, adjusted baseline amplitude and peak latency were measured for all 3 components in both modalities. Adjusted baseline amplitude measures were calculated at Cz from peak amplitudes relative to the two adjacent peaks of opposite polarity (D'Arcy et al., 2011; Ghosh-Hajra et al., 2016a). All peaks were obtained with a semi-automatic process using Brain Vision Analyzer, within expected latency windows, identifying local peak amplitudes (as defined by Luck, 2014, p. 285) of expected polarity (Marchand et al., 2002). Latency windows vary across studies, depending on stimulus types, task conditions, subject age, etc. (Polich and Kok, 1995; Polich, 1997; Cano et al., 2009). Hence it is recommended to choose latency windows based on both literature and visual inspection of the GA waveforms (Cassidy et al., 2012; Chronaki et al., 2012; Pfabigan et al., 2014; López Zunini et al., 2016). Due to the wide range of age (19-66yrs) and two modalities within this study, latency windows for each component were chosen according to several previous studies. For both modalities, the N100 peak, was measured between 75 and 200 ms (Johannes et al., 1995; Covington and Polich, 1996; Niznikiewicz et al., 1997; Hillyard and Lourdes, 1998; Knott et al., 2003; Huang et al., 2011). Shorter latencies were used for P300 in auditory (250-500ms) compared to visual (250–600 ms) (Comerchero and Polich, 1998; Bernat et al., 2001; Knott et al., 2003; Cano et al., 2009; Tacikowski and Nowicka, 2010; Campanella et al., 2012). The latency window for N400 peaks was 300–650 ms for auditory and visual (Marchand et al., 2002; D'Arcy et al., 2003; Kutas and Federmeier, 2011).

EBS results comprised of six total ERP measures (3 components × 2 measures), generated through a linear transformation. Each measure, amplitude or latency values, were normalized and ranked from 0 to 1 based on the normative group mean and the best possible outcome following the methods as shown before in Fickling et al. (2018) and (Ghosh-Hajra et al., 2016a). The normative group used was the subjects recruited in this study, separate for each modality. Mathematically, EBS measures can be expressed as shown in Equations (1, 2) below:

Score=1-abs [(M-best)/(max-min)]
Score=1-abs [(best-M)/(max-min)]

The M represents the mean value of either the amplitude or latency. The max and min are the maximum value and the minimum value, respectively. The best variable is the “ideal” value that should be achieved, which can either be the max or the min value depending on whether the lowest or the highest value represents the ideal situation. For instance, an “ideal” value for latency is generally shorter because it represents faster (better) processing, whereas for amplitude values, depending on the targeted ERP component, the highest positive value or lowest negative value is thought to represent “ideal” processing (Ghosh-Hajra et al., 2016a). Both larger amplitudes and shorter latencies translate to higher EBS scores. Equation (1) is utilized for N100 and N400 amplitude and latency as well as P300 latency, whereas Equation (2) is used for P300 amplitude. This translation allows for complex ERP data to become accessible metrics, while preserving the underlying ERP results. This technique also will enable normalization within modalities to account for the known differences while preserving the relationship across modalities.

Adjusted baseline amplitude and peak latency values, as well as EBS values were compared at the group-level across modalities using JMP (JMP®, Version 12.2.0 SAS Institute Inc., Cary, NC). Normality was assessed using the Shapiro-Wilk W test. Normality was assessed using the Shapiro-Wilk W test. Only the measures for visual P300 amplitude did not pass the normality test, therefore the Wilcoxon test was used for comparison. All others were compared using matched pairs *t*-test. Results are presented as mean ± SD.

Pearson correlation coefficient (Pearson r) was used to evaluate the relationship between individual values across modalities. This statistic assumes a linear relationship and is confirmed by inspection of the r-value, associated p-value and scatter plot. Pearson R correlation analysis was used for all except P300 amplitude values. The visual P300 amplitude values failed the Shapiro-Wilk test of normality (i.e., non-parametric distribution) so Spearman rho was used for correlation analysis.

## Results

### Targeted ERP Responses

#### Mean Amplitude Analysis

**Figure 2:**
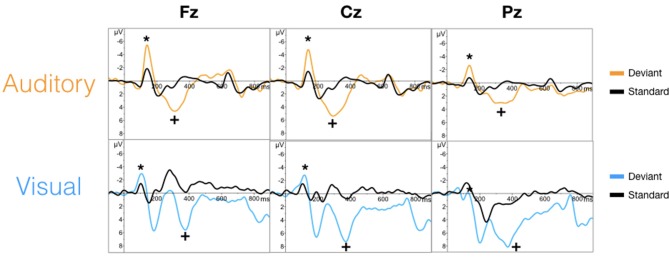
Grand averaged waveforms for the N100 (^*^) and P300 (+) component in auditory **(top)** and visual **(bottom)** modalities.

**Figure 3:**
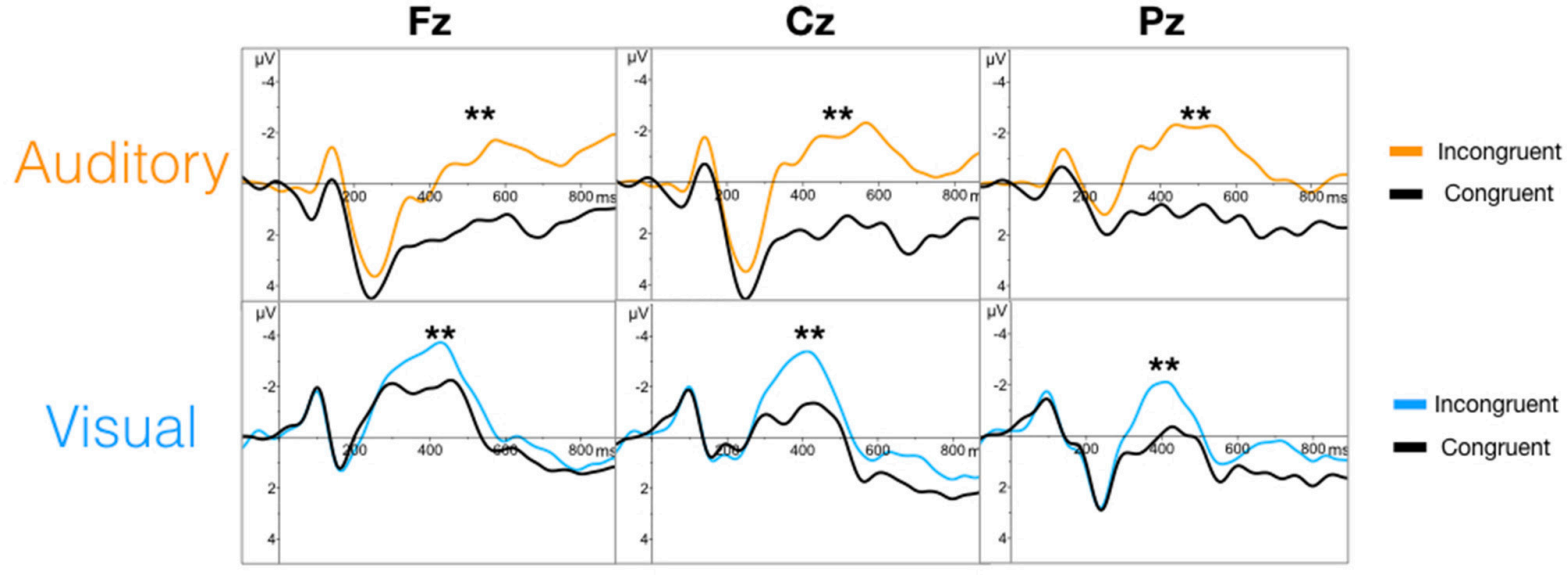
Grand averaged waveforms for the N400 (^**^) in the auditory **(top)** and visual **(bottom)** modalities.

The targeted N100 and P300 components were successfully evoked using oddball paradigms within the auditory and visual sequences (Figure 2). Similarly, the targeted N400 component was evoked by the word pair paradigm within the auditory and visual sequences (Figure 3).

ANOVAs for the mean amplitudes within each modality revealed main effects for stimulus type across all three components, with no interaction effect found. Tables 1, 2 provide quantitative mean amplitude measures for group-level N100, P300, and N400. Table 3 provides a summary of ANOVA effects tests. For box plots illustrating the difference in mean amplitudes for each condition and ERP for both modalities, see Supplementary Figures 1–3.

**Table 1:** Summary Statistics: Mean amplitude measures for group-level N100 and P300 (μV).

**ERP**	**Channel**	**Auditory oddball stimulus (μV)**		**Visual oddball stimulus (μV)**	
		**Standard**	**Deviant**	**Standard**	**Deviant**
N100	Fz	−1.46 ± 1.84	−4.09 ± 2.69	−0.90 ± 2.12	−2.60 ± 2.63
	Cz	−0.97 ± 1.45	−3.42 ± 2.29	−1.05 ± 2.20	−2.27 ± 2.44
P300	Fz	0.01 ± 1.06	2.81 ± 2.50	−1.22 ± 3.03	2.57 ± 4.62
	Cz	0.22 ± 0.94	3.40 ± 2.34	−0.12 ± 3.02	4.74 ± 4.28
	Pz	0.21 ± 0.64	2.42 ± 2.15	1.01 ± 2.10	5.81 ± 3.61

*Mean ± SD*.

**Table 2:** Summary Statistics: Mean amplitude measures for group-level N400 (μV).

**ERP**	**Channel**	**Auditory word pair stimulus (μV)**		**Visual word pair stimulus (μV)**	
		**Congruent**	**Incongruent**	**Congruent**	**Incongruent**
N400	Cz	4.26 ± 4.47	−1.15 ± 4.62	−1.28 ± 6.92	−2.88 ± 7.04
	Pz	2.31 ± 3.18	−2.27 ± 3.45	1.58 ± 5.67	−0.25 ± 6.13

*Mean ± SD*.

**Table 3:** Summary of the Effects Tests: *F*-ratio and *p*-values of all the main effects and interaction effects of mean amplitude ANOVAs.

**ERP**	**Source**	**Auditory**		**Visual**	
		**F Ratio**	**Prob > F**	**F Ratio**	**Prob > F**
N100	Stimulus	**78.4661**	**<0.0001**	**29.8380**	**<0.0001**
	Channel	3.8962	0.0516	1.0253	0.3142
	Stimulus^*^ Channel	0.0907	0.7640	1.3884	0.2420
P300	Stimulus	**137.0415**	**<0.0001**	**138.8442**	**<0.0001**
	Channel	1.7835	0.1717	**17.2778**	**<0.0001**
	Stimulus^*^ Channel	1.4747	0.2323	0.8177	0.4435
N400	Stimulus	**86.3009**	**<0.0001**	**6.8476**	**0.0105**
	Channel	**8.1326**	**0.0054**	**17.6354**	**<0.0001**
	Stimulus^*^ Channel	0.5831	0.4471	0.0327	0.8570

*Significance of <0.05 is denoted with bold text*.

### Comparison and Normalization of Auditory and Visual Sequences

#### Adjusted Baseline Amplitude and Peak Latency Measures

Table 4 provides group averaged adjusted baseline amplitude and peak latency measures for the 3 components across modalities. There was no significant difference for amplitude in either the N100 and P300. However, the N400 amplitudes showed a significant difference between auditory (−5.82 ± 2.11 μV) and visual (−6.82 ± 1.80 μV) modalities (*p* = 0.0061). As expected, all three ERP components showed significant latency differences. For a bar-graph illustrating the adjusted baseline amplitude and latency measures pairwise comparisons (matched pairs *t*-tests) across modalities, please see Supplementary Figures 4 and 5.

**Table 4:** Summary Statistics: adjusted baseline amplitude and peak latency measures for group-level ERP characteristics at Cz.

**ERP**	**Measure**	**Auditory**	**Visual**	***P*-value**
N100	Amplitude (μV)	−9.17 ± 3.12	−8.80 ± 3.26	0.8089
	Latency (ms)	**139.33** **±** **10.60**	**123.13** **±** **21.43**	**0.0009**
P300	Amplitude (μV)	8.06 ± 3.79	8.87 ± 2.63	0.5040
	Latency (ms)	**309.00** **±** **42.82**	**369.07** **±** **58.56**	***p*** **<** **0.0001**
N400	Amplitude (μV)	**−5.82** **±** **2.11**	**−6.82** **±** **1.80**	**0.0061**
	Latency (ms)	**488.73** **±** **58.97**	**414.40** **±** **30.47**	***p*** **<** **0.0001**

*Mean ± SD. Significance of <0.05 is denoted with bold text*.

#### Elemental Brain Scores (EBS)

**Figure 4:**
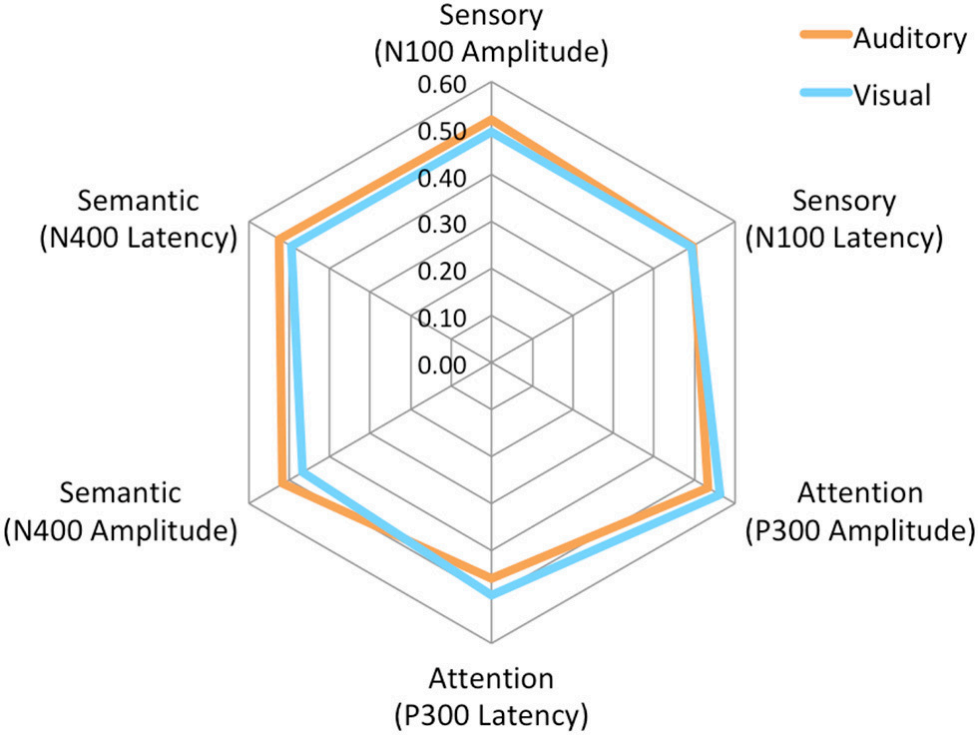
Radar Plot of amplitude and latency EBS values for both modalities across all 3 ERP components.

No significant differences were found for any comparisons using the mean EBS in matched pairs *t*-tests (see Table 5). Auditory and visual group EBS in all 6 measures results are also depicted visually (see Figure 4).

**Table 5:** Elemental Brain Scores (EBS) measures for group-level ERP characteristics.

**ERP**	**Measure**	**Auditory scores**	**Visual scores**	***P*-value**
N100	Amplitude (μV)	0.52 ± 0.17	0.49 ± 0.17	0.4491
	Latency (ms)	0.49 ± 0.17	0.49 ± 0.17	0.9343
P300	Amplitude (μV)	0.53 ± 0.17	0.56 ± 0.17	0.1818
	Latency (ms)	0.46 ± 0.16	0.50 ± 0.17	0.2629
N400	Amplitude (μV)	0.52 ± 0.17	0.47 ± 0.17	0.0995
	Latency (ms)	0.53 ± 0.17	0.50 ± 0.17	0.4279

*Mean ± SD within-subject elemental brain scores across modalities*.

#### Correlation Analysis

**Figure 5:**
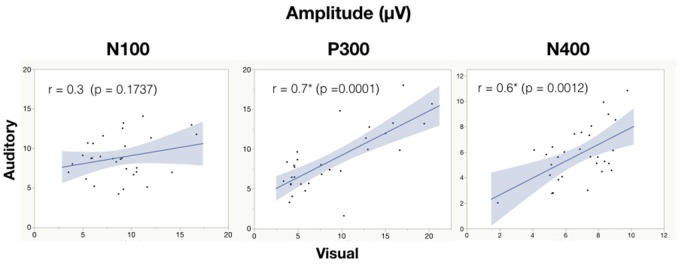
Correlation analysis between auditory and visual adjusted baseline amplitude values for each subject. Significance of < 0.05 is denoted with ^*^.

See Table 6 for all correlations and Figure 5, 6 for amplitude and latency scatter plots. Moderate to high correlations were found across modalities in amplitude for P300 (rho = 0.7, *p* = 0.0001) and N400 (*r* = 0.6, *p* = 0.0012) and P300 latency (*r* = 0.5, *p* = 0.0033). The N100 amplitude and latency, and N400 latency showed no significant correlations.

**Table 6:** Correlations of amplitude and latency measures at Cz.

**ERP**	**Measure**	**Correlation (r)**	***p*-value**
N100	Amplitude (μV)	0.3	0.1737
	Latency (ms)	0.04	0.8470
P300	Amplitude (μV)	0.7^*^	**0.0001**
	Latency (ms)	**0.5**	**0.0033**
N400	Amplitude (μV)	**0.6**	**0.0012**
	Latency (ms)	0.2	0.3135

Pearson r correlation coefficient used for all normally distributed data and Spearman rho used for non-parametric data, P300 amplitude.

* *Significance of <0.05 is denoted with bold text*.

**Figure 6:**
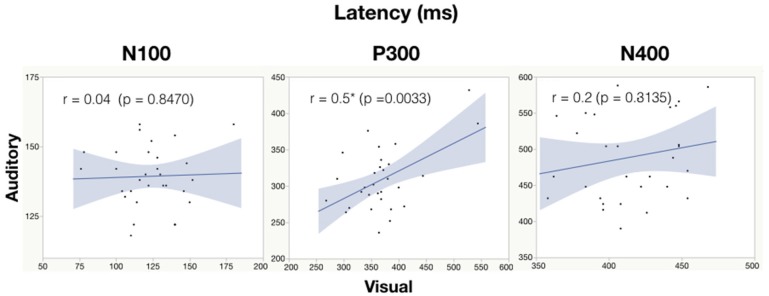
Correlation analysis between auditory and visual peak latency values for each subject. Significance of < 0.05 is denoted with ^*^.

## Discussion

The current study had two objectives: (1) Translate the interlaced, rapid auditory sequence into a visual sequence and validate it by assessing if the targeted EPRs (N100, P300, and N400) are successfully evoked in a healthy population; and (2) Compare the ERP responses (amplitudes and latencies) between visual and auditory modalities, and evaluate the relationship between modalities within individuals.

### Objective 1: Targeted ERP Responses

As an initial validity check, the results demonstrated that the targeted ERPs (N100, P300, and N400) were evoked and detectable by comparing mean amplitudes for each stimulus conditions within each modality at a group-level. As expected, significant conditional differences were found for the N100, P300, and N400 responses for both auditory and visual modalities (Tables 1–3). Within the visual modality, the increased N100 amplitude to the contrast change is consistent with past studies using similar stimuli (Dustman et al., 1982; Johannes et al., 1995; Covington and Polich, 1996; Carrillo-de-la-Peña et al., 1999). The increased P300 amplitude to viewing one's own name further was consistent with the allocation of information processing resources associated with self-relevant information (Müller and Kutas, 1996; Herzmann et al., 2004; Perrin et al., 2005; Herzmann and Sommer, 2007; Polich, 2007; Zhao et al., 2009, 2011; Tacikowski and Nowicka, 2010; Cygan et al., 2014; Sculthorpe-Petley et al., 2015). Similarly, larger visual N400 amplitudes to incongruent word stimuli was due to increased processing in response to violations of semantic expectancies (Rugg, 1985; Brown and Hagoort, 1993; Osterhout and Holcomb, 1996; Chwilla et al., 1998; Brown et al., 2000; Lau et al., 2008; Kutas and Federmeier, 2011; Ghosh-Hajra et al., 2016a).

Effects of channel location differed across the two modalities for the N400 response. On average larger mean amplitudes were found at Cz compared to Pz for the auditory presented words [*p* = 0.0054, estimated mean difference = 1.54 μV (*SE* = 0.54)]. Whereas for the visually presented words, slightly larger estimated means were found at Pz compared to Cz [*p* < 0.0001, estimated mean difference = 2.75 μV (*SE* = 0.65)]. Despite the on average larger mean amplitudes measured at Pz for visual words across stimulus conditions, the difference between congruent and incongruent conditions is of interest when establishing the N400 effect. Further *post-hoc* analysis showed the estimated mean difference between stimulus conditions at the two electrodes was only slightly larger at Pz (1.83 μV, *SE* = 0.26) compared to Cz (1.59 μV, *SE* = 0.26). Despite the small difference between Cz and Pz, the N400 effect was still measurable at Cz, which is the site used in past brain vital signs research and the site chosen for modality comparison in this study (Ghosh-Hajra et al., 2016a; Fickling et al., 2018). The results are in line with previous literature, with the N400 effect typically being measured at midline centro-parietal scalp sites (Kutas et al., 1987; Kutas and Federmeier, 2011; Kutas and Hillyard, 1982; van Petten and Rheinfelder, 1995).

### Objective 2: Comparison and Normalization of Auditory and Visual Sequences

As expected, there were significant modality-related latency differences for all three components (see Table 4). The only difference in ERP activation (at Cz) was a significant increase in amplitude of the visual N400. However, the standardized conversion of all three ERP components into EBS allowed for normalization of both response latencies and amplitudes, with no significant difference (see Figure 4 and Table 5). The translation into EBS, however, did not affect the correlation across modalities within individuals because the linear translation from ERP measures to EBS are calculated only relative to the normative database (*N* = 30) within each modality separately, therefore not affecting the relationship across modalities. Correlations done with EBS and ERP measures were identical. Correlation analysis showed significant, moderate to strong (0.5–0.7) correlations for amplitude measures for P300 amplitude and latency as well as N400 amplitude across modalities (see Table 6 and Figures 5, 6). The combination of all these results and comparison between modalities across the targeted ERP components has given us initial insight into the relationship between modalities.

The N100 is typically reported with earlier peak latencies for the auditory modality (Niznikiewicz et al., 1997; Knott et al., 2003), however this trend was reversed in the current results, which was likely due to increasing the intensity contrast between black and white stimuli (Dustman et al., 1982; Carrillo-de-la-Peña et al., 1999). Significant group-level differences and non-significant correlations for sensory (N100) latencies between modalities suggest that speed in sensory processing differs and is not predictive within individuals across modalities. The lack of correlation between the auditory and visual N100 amplitudes at Cz possibly reflects that inconsistent levels of sensory processing were being evoked by the auditory and visual stimuli within individuals. Further analysis of the location of the max N100 amplitude for each modality is needed.

It is notable that the P300 results arose from two very different manipulations; no significant difference was found at the group-level and a strong correlation of adjusted baseline amplitude between modalities was found (rho = 0.7, *p* = 0.0001; Table 6). These results imply that similar levels of attention allocation (marked by P300 activation) were being evoked within individuals from either sequence despite the different oddball approaches. Given that the P300 is produced by a distributed network of brain processes associated with attention and memory operations (Polich, 2007), the visual P300 latency delay found was likely related to more complex information processing required for visual identification of SON versus a simple auditory deviant tone (Kramer et al., 1986; Verleger, 1997; Halgren et al., 1998; Bennington and Polich, 1999; Patel and Azzam, 2005; Polich, 2007; Duncan et al., 2009). Based on past literature and the correlated (*r* = 0.5; *p* = 0.0033) but differing group-level peak latencies (*p* < 0.0001) found, it can be concluded that similar functional processes of attention were evoked with a possible systematic difference of modalities, where the visual deviant stimulus requires slightly longer time for detection and processing compared to the auditory deviant stimulus. The correlation also implies that the individual relative speed of detection and classification of the deviant stimuli was similar across modalities; reflecting that attention processing speed within an individual is similar regardless of the stimulus modality.

The visual deviant condition was primarily used to evoke a sensory response (N100), however, it was presented in combination with the SON. It was chosen in order to reach our first objective of developing a passive visual sequence that successfully evokes the targeted ERP responses. This salient stimulus may have affected the P300, however, such a change in brightness has been documented to elicit an early N100 response and a P200 prior to the P300 (Hruby and Marsalek, 2003; Dustman et al., 1982; Carrillo-de-la-Peña et al., 1999). These early visual sensory (N100-P200) responses often occur with P300 components in visual oddball paradigms and should not have interfered with the P300 evoked from participants recognizing their own names. The stimulus was presented for 600 ms, allowing plenty of time for participants to react and adjust to the contrast change and recognize their names. The change in contrast may have caused participants to increase their engagement in the task and level of attention to when their names were presented, in turn potentially affecting the magnitude (amplitude) of the P300 response to the SON. However, the visual oddball paradigm used appeared to be evoking similar levels of attentional responses as the auditory paradigm within individuals; no significant difference at the group-level and a strong correlation of adjusted baseline amplitude between modalities was found (rho = 0.7, *p* = 0.0001). These results imply that similar levels of attention allocation (marked by P300 activation) were being evoked in subjects from either sequence despite the different oddball approaches. Future work could be done to compare SON without a contrast flip to see the impact on the P300 response and if there is a confounding effect.

In spite of being modality independent, aspects of the N400 have been found to differ across visual and auditory processing of words (Kutas and Hillyard, 1980; McCallum et al., 1984; Bentin et al., 1985; Kutas et al., 1987; Holcomb and Neville, 1990; Kutas and Federmeier, 2011). In general, the auditory N400 tends to be characterized by a lower amplitude, later peak, and longer duration response (Kutas and Federmeier, 2011). This pattern was reflected in our results and, despite the significant amplitude differences, was notably equated by the EBS transformation and showed a significant moderate correlation of amplitude (*r* = 0.6, *p* = 0.0012). These results imply that the modality amplitude difference is possibly systematic; a similar level of semantic processing relative to each modality is being evoked within individuals across modality paradigms.

Emerging neuroimaging technologies have allowed for further investigation into theories of early word processing and recognition (Carreiras et al., 2014). Competing theories still debate on the precise initial recognition process of printed and spoken words, however, data shows that both reading and listening are incremental and largely a serial processes (Rayner et al., 2009; review by Carreiras et al., 2014). Nevertheless, reading (visual linguistic processing) is faster than listening (auditory linguistic processing) (Breznitz and Berman, 2003), with reading able to reach relatively high speeds (250–350 wpm for most skilled readers) not thought achievable for listening comprehension (Rayner et al., 2009). This difference in speed between reading and listening processing is reflected in ERP studies, with shorter latencies and durations typical of a visual N400 relative to an auditory N400 (Holcomb et al., 1992; Kutas and Federmeier, 2011; Luck, 2005). This may account for the differing latencies we found across modalities. Furthermore, the lack of correlation in latency also implies that fast reading ability is not predictive of fast speech comprehension and vice versa. Individual differences may have been a factor; for instance, some participants may have stronger reading skills than auditory comprehension skills.

Overall, our analyses demonstrated a clear pattern of results that supported the concept of visual brain vital signs. Specifically, the results confirmed the following observations: (1) All three visual components were measurable at central electrode locations, showing potential for portable EEG application in the future, as done with previous brain vital signs studies (Ghosh-Hajra et al., 2016a; Fickling et al., 2018); (2) Overall modality comparison analysis at the central electrode site (Cz) revealed that primarily attention (P300), as well as semantic (N400) processing, are potentially transferrable and comparable across modalities, however sensory (N100) processing is not; and (3) it was possible to show that the brain vital sign framework can be implemented in visual modality format in order to facilitate clinical applications where this is necessary, such as cognitive impairment in aging populations with hearing loss (Lin et al., 2013).

### Limitations

Within the modality comparison analysis, the current study focused largely on temporal component differences in terms of response amplitudes and latencies (at Cz). It did not evaluate spatial distribution differences and/or source localization differences—for which there would be full expectation of underlying neuroanatomical differences that cannot/should not be standardized. Future studies will better characterize boundary limits for spatial overlap. (For initial exploratory analysis see Supplementary Figures 10–15, which illustrate topographical maps using CSD for each ERP component in each modality.) Aspects of the EEG analysis, such as the reference chosen may affect further analysis. The linked mastoid reference was chosen after careful consideration for this study; however, referencing methods have limitations because a truly neutral point on the body is impossible. Other referencing methods such as the reference electrode standardization technique (REST) provide a reference of scalp EEG recordings to a point at infinity (Yao, 2001; Dong et al., 2017). Initial exploratory analysis of REST was undertaken (see Supplementary Figures 6–9). Further comparison analysis of references will be done in the future. Another important limitation relates to the need for separate patient/clinical validation studies for visual brain vital signs to replicate the auditory modality results in concussion, aging, and dementia. That is, the assumption cannot be made that a common pattern of results exists for a specific condition (e.g., dementia). Instead, it will be important to conduct similar comparison based studies for particular neurological conditions and characterize the relationship of results across modalities. However, comparisons across modalities will likely be an important feature of brain vital sign monitoring in terms complex issues related to diagnostic sensitivity and specificity. For instance, in the case of dementia, it can help discriminate age-related hearing loss vs. the detection of cognitive impairment.

## Conclusion

The current study reinforced the viability of the brain vital sign framework through successful expansion from the auditory to the visual modality. Despite some modality differences found, comparison analysis showed that modality differences can be standardized within EBS results, and that attentional and language processing are potentially transferrable between modalities. Visual modality brain vital signs provide an important alternative, particularly for populations in which monitoring cognitive function changes may be complicated by hearing loss (e.g., elderly and dementia). Further investigation into modality differences should examine spatial distribution differences together with comparison validation studies for specific neurological conditions like dementia. Nonetheless, with visual brain vital signs added to the overall framework it is possible to expand clinical applications and provide further insight into point-of-care monitoring of brain function.

## Ethics Statement

This study was carried out in accordance with the recommendations of Ethical Conduct for Research Involving Humans (the TCPS-2), Research Ethics Boards at Simon Fraser University and Fraser Health Authority with written informed consent from all subjects. All subjects gave written informed consent in accordance with the Declaration of Helsinki. The protocol was approved by the Research Ethics Boards at Simon Fraser University and Fraser Health Authority.

## Author Contributions

All authors contributed to conceptualization and study design. GP, SG-H, CL, SF, and RD: literature search. GP, SG-H, CL, and SF: data collection. SG-H, CL, SF, SR, and RD: analysis planning. GP: data analysis. GP, SG-H, CL, SF, XS, and RD: result presentation. GP, SG-H, CL, SF, XS, and RD: analysis outcome verification. All authors contributed to result interpretation, manuscript preparation and editing and approved the final draft. This publication is the original work of the authors and RD will serve as guarantor of its contents.

### Conflict of Interest Statement

One of the authors (RD) is associated with HealthTech Connex Inc. which may qualify them to financially benefit from the commercialization of a NeuroCatch™platform capable of measuring brain vital signs. The remaining authors declare that the research was conducted in the absence of any commercial or financial relationships that could be construed as a potential conflict of interest.
