# Supplementary material for: Brain Vital Signs: Expanding From the Auditory to Visual Modality
Source: Front Neurosci. 2019 Jan 18;12:968. doi: 10.3389/fnins.2018.00968 (PMC6346702; doi:10.3389/fnins.2018.00968)
Supplement: Supplementary file 1 [file Table_1.docx]

Supplementary Material

Brain vital signs: Expanding from the auditory to visual modality

Gabriela M. Pawlowski 1,2*, Sujoy Ghosh-Hajra1,3, Shaun D. Fickling1,3, Careesa C. Liu1,3, Xiaowei Song1,3, Stephen Robinovitch2, Sam M. Doesburg2 and Ryan C. N. D’Arcy 1,2,3*

1 NeuroTech Laboratory, Faculty of Applied Sciences, Simon Fraser University, Metro Vancouver, BC, Canada, 2 Biomedical

Physiology and Kinesiology, Faculty of Science, Simon Fraser University, Metro Vancouver, BC, Canada, 3 Health Sciences

and Innovation, Surrey Memorial Hospital, Fraser Health Metro Vancouver, BC, Canada

*** Correspondence**: Gabriela Pawlowski, MSc. ([gabriela_pawlowski@sfu.ca](mailto:gabriela_pawlowski@sfu.ca)); Ryan C.N. D’Arcy, Ph.D. (ryan_darcy@sfu.ca)

# Supplementary Figures


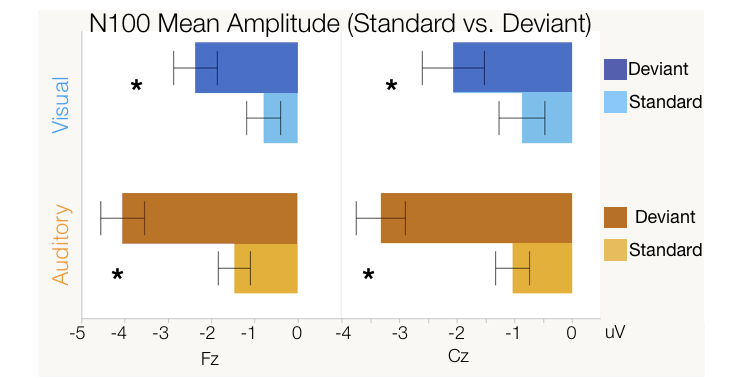


Supplementary Figure 1. Box Plots illustrating the difference between standard and deviant stimuli conditions in auditory (orange) and visual (blue) N100 mean amplitude ANOVA analysis. Significance of < 0.05 is denoted with *.


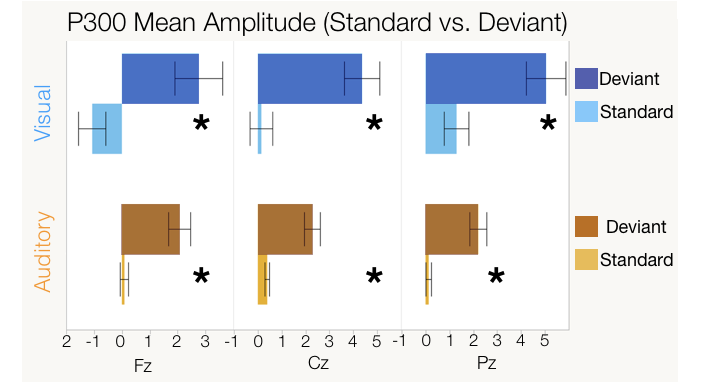


Supplementary Figure 2. Box Plots illustrating the difference between standard and deviant stimuli conditions in auditory (ornage) and visual (blue) P300 mean amplitude ANOVA analysis. Significance of < 0.05 is denoted with *.


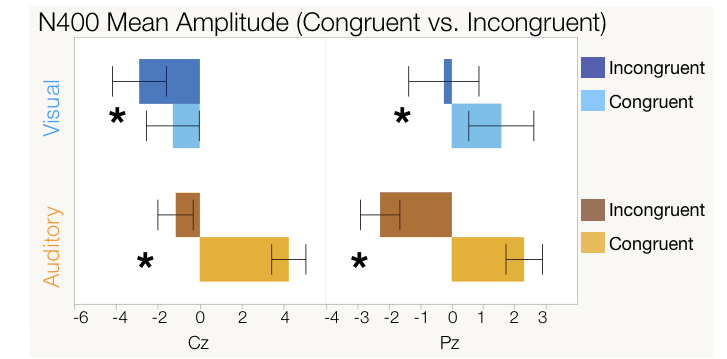


Supplementary Figure 3. Box plots illustrating the difference between congruent and incongruent word pair stimuli conditions in auditory (orange) and visual (blue) N400 mean amplitude ANOVA analysis. Significance of < 0.05 is denoted with *.


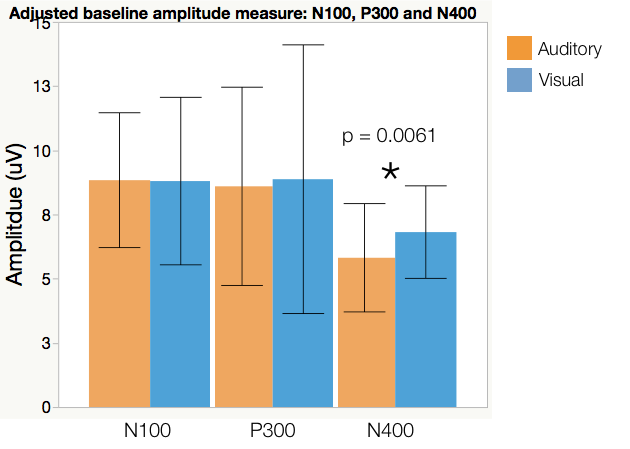


Supplementary Figure 4. Pairwise comparisons (matched pairs t-tests) of adjusted baseline amplitude measures in auditory (orange) and visual (blue), showing the difference between modalities. Significance of < 0.05 is denoted with *.


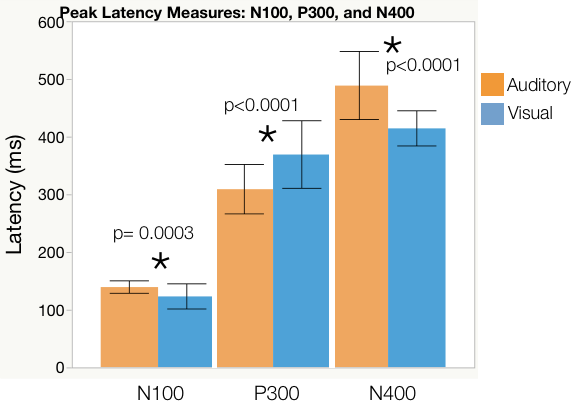


Supplementary Figure 5. Pairwise comparisons (matched pairs t-tests) of peak latency measures in auditory (orange) and visual (blue), showing the difference between modalities. Significance of < 0.05 is denoted with *.


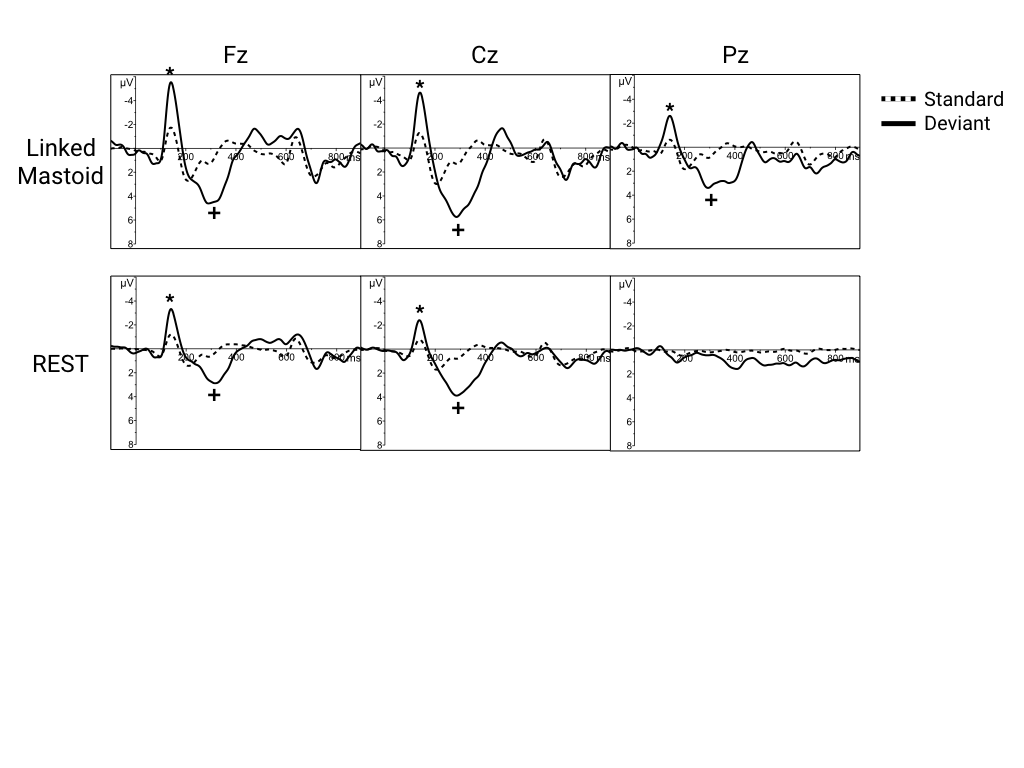


Supplementary Figure 6. Grand average waveforms for auditory N100 (*) and P300 (+) components using linked mastoid (top) and REST (bottom) referencing techniques.


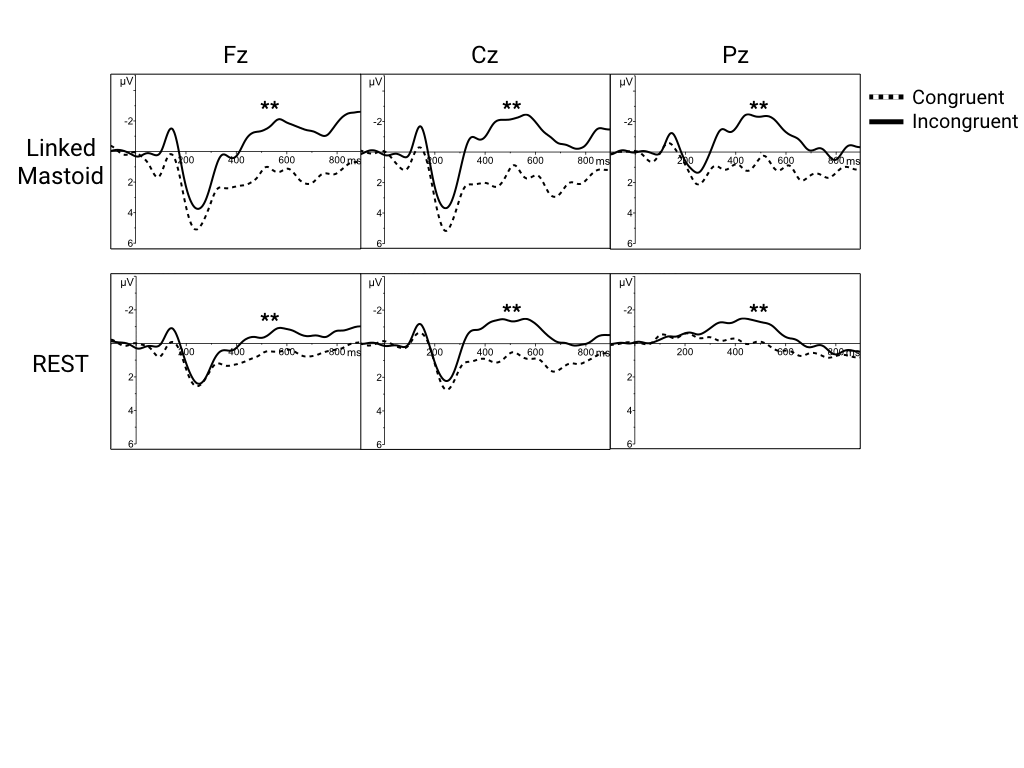


Supplementary Figure 7. Grand average waveforms for auditory N400(**) component using linked mastoid (top) and REST (bottom) referencing techniques.


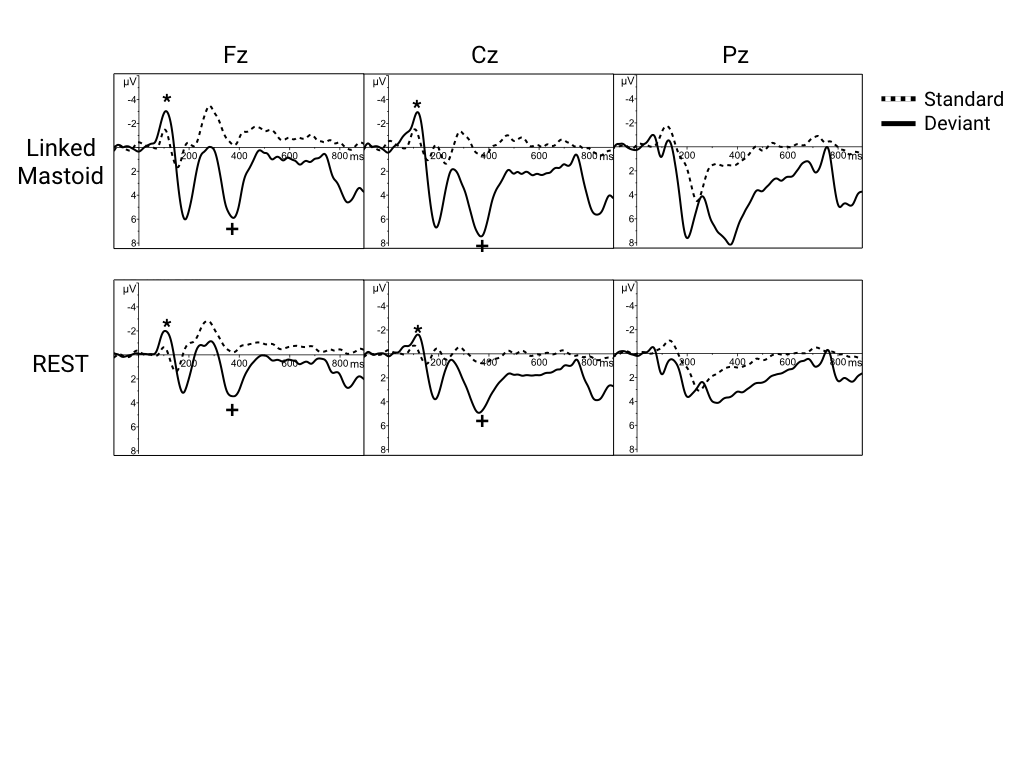


Supplementary Figure 8. Grand average waveforms for visual N100 (*) and P300 (+) components using linked mastoid (top) and REST (bottom) referencing techniques.


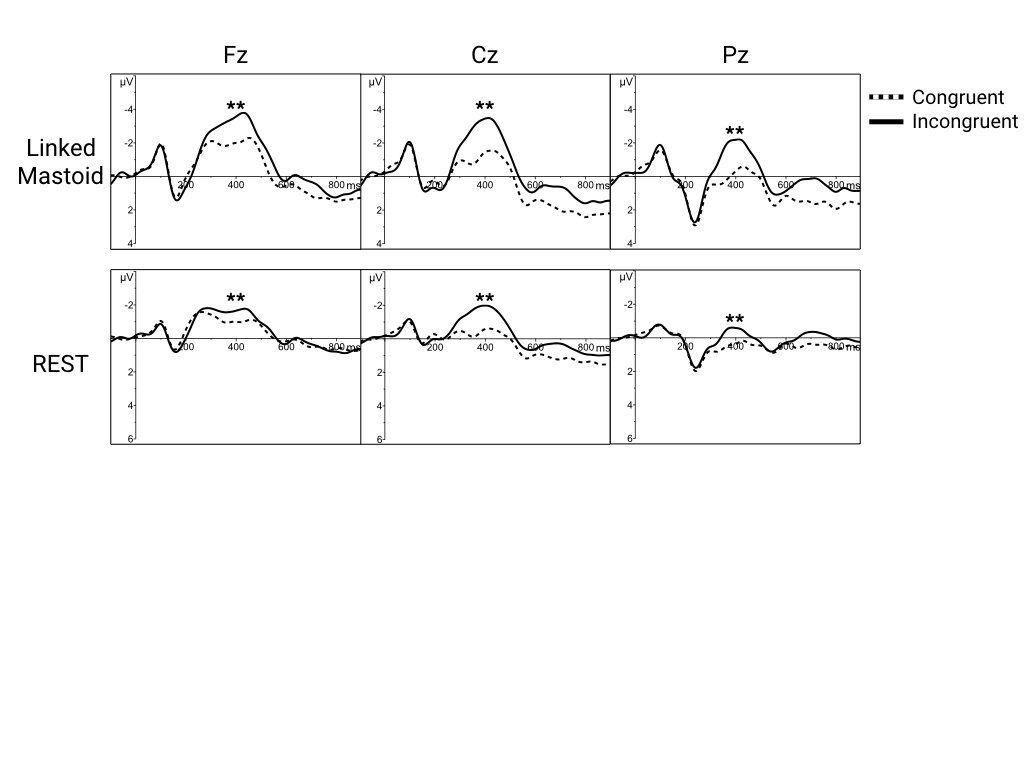


Supplementary Figure 9. Grand average waveforms for visual N400(**) component using linked mastoid (top) and REST (bottom) referencing techniques.

Supplementary Figure 10. Auditory N100 topographical map. The CSD map used 4th order splines, with a max legendre polynomial degree of 10, based on a defaul lambda of 1e-5.


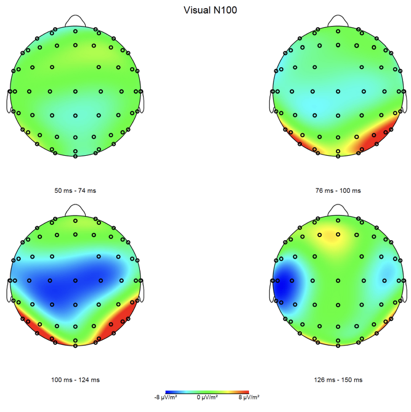


Supplementary Figure 11. Visual N100 topographical map. The CSD map used 4th order splines, with a max legendre polynomial degree of 10, based on a defaul lambda of 1e-5.


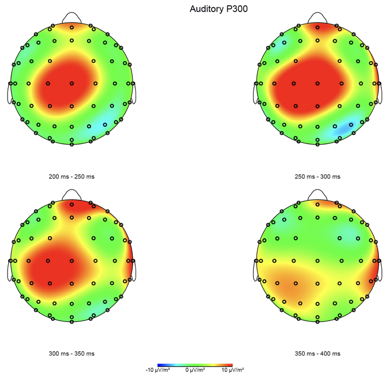


Supplementary Figure 12. Auditory P300 topographical map. The CSD map used 4th order splines, with a max legendre polynomial degree of 10, based on a defaul lambda of 1e-5.


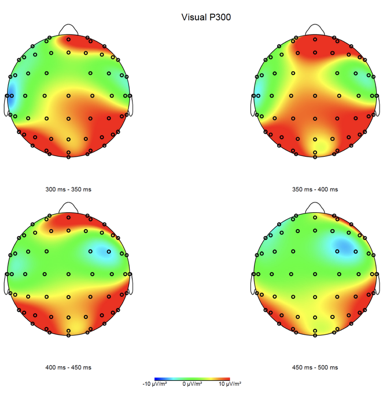


Supplementary Figure 13. Visual P300 topographical map. The CSD map used 4th order splines, with a max legendre polynomial degree of 10, based on a defaul lambda of 1e-5.


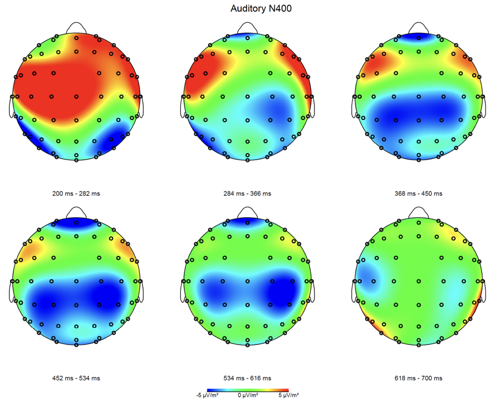


Supplementary Figure 14. Auditory N400 topographical map. The CSD map used 4th order splines, with a max legendre polynomial degree of 10, based on a defaul lambda of 1e-5.


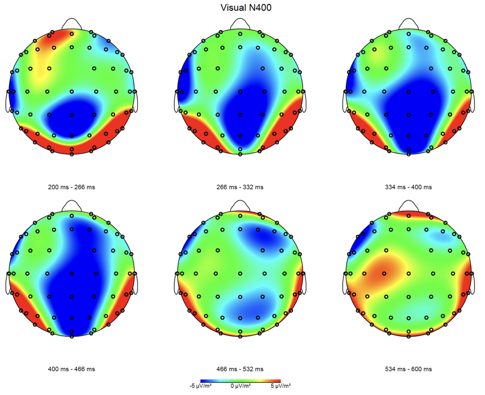


Supplementary Figure 15. Visual N100 topographical map. The CSD map used 4th order splines, with a max legendre polynomial degree of 10, based on a defaul lambda of 1e-5.
